# Cooperating to show that you care: costly helping as an honest signal of fitness interdependence

**DOI:** 10.1098/rstb.2020.0292

**Published:** 2021-11-22

**Authors:** Pat Barclay, Rebecca Bliege Bird, Gilbert Roberts, Szabolcs Számadó

**Affiliations:** ^1^ Department of Psychology, University of Guelph, 50 Stone Road E., Guelph, ON, Canada, N1G 2W1; ^2^ Department of Anthropology, Pennsylvania State University, PA, USA; ^3^ Independent Researcher, Budapest University of Technology and Economics, Hungary; ^4^ Department of Sociology and Communication, Budapest University of Technology and Economics, Hungary; ^5^ Center for Social Sciences, Eötvös Loránd Research Network (ELKH), Hungary

**Keywords:** costly signalling, stake, trust, food sharing, altruism, welfare trade-off ratio

## Abstract

Social organisms often need to know how much to trust others to cooperate. Organisms can expect cooperation from another organism that depends on them (i.e. stake or fitness interdependence), but how do individuals assess fitness interdependence? Here, we extend fitness interdependence into a signalling context: costly helping behaviour can honestly signal one's stake in others, such that those who help are trusted more. We present a mathematical model in which agents help others based on their stake in the recipient's welfare, and recipients use that information to assess whom to trust. At equilibrium, helping is a costly signal of stake: helping is worthwhile for those who value the recipient (and thus will repay any trust), but is not worthwhile for those who do not value the recipient (and thus will betray the trust). Recipients demand signals when they value the signallers less and when the cost of betrayed trust is higher; signal costs are higher when signallers have more incentive to defect. Signalling systems are more likely when the trust games resemble Prisoner's Dilemmas, Stag Hunts or Harmony Games, and are less likely in Snowdrift Games. Furthermore, we find that honest signals need not benefit recipients and can even occur between hostile parties. By signalling their interdependence, organisms benefit from increased trust, even when no future interactions will occur.

This article is part of the theme issue ‘The language of cooperation: reputation and honest signalling’.

## 1. Introduction

Humans are intensely social mammals whose fitnesses are often highly interdependent, i.e. one person's fitness can positively impact another's. This interdependence arises from a number of sources: kinship both real and symbolic; dyadic cooperative partnerships in sharing, hunting, childcare and divisions of labour; and the passive or emergent benefits of living in a social group, such as predator or resource defence, information sharing and social support; this interdependence is widely argued to facilitate many forms of active cooperation (e.g. [1–9]). However, it is not always clear how available group members will be in the future or how interdependent two individuals are—just because A has a high stake in the success of B and plans for future cooperation, it does not mean that B feels the same way about A, or knows how A feels. Thus, individuals living in flexible social groups face a significant problem: how to communicate the strength of that interdependence to other members to ensure trust and keep groups from falling apart. To do this, individuals need to determine whom to trust, and how (and whether) to honestly communicate how much they value others in order to be trusted in return.

Many social relationships face the problem of signalling interdependence to build trust, but it is particularly relevant in small communities where subsistence work requires extensive cooperation. For example, among Martu, Aboriginal hunters in Australia's Western Desert, women are avid small-animal hunters who frequently form ‘dinner-camps’—small foraging groups that include foragers and non-foragers of mixed sex and age and sleeping residency [10–12]. These foraging groups travel to a cooking hearth, disperse to hunt and gather for several hours, then return to the hearth to process, cook and share the food. After the meat has been cooked, the hunter first divides her harvest with her hunting partner(s), and then each hunter distributes meat to others present, to ensure that everyone has an approximately equal portion of meat [11,13]. This means that the hunter often takes home only a small portion of her own harvest, and better hunters end up giving away a much higher proportion of their harvest than poorer hunters. Because hunters are not favoured recipients of meat according to how much they donated to the common pot, better hunters end up contributing to the collective good at a cost to themselves [12].

Martu women explain that better hunters benefit not from the meat consumed, but from the friendships generated through generous sharing. They say that a major dilemma in cooperative hunting is finding a partner who will split the catch evenly with them in the first round of sharing. And indeed, women who are better hunters typically share more, and those who share more generously on average tend to pair up with other generous hunting partners. Thus, even though a woman does not keep the meat she splits with her partner, women gain social status from generosity, because giving meat away generates goodwill regardless of who killed it.

While initial analysis suggested that cooperative hunting partnerships were one benefit of costly sharing [11], the women themselves are adamant that the benefits go well beyond this to the formation of *pukurrpa*, the happiness and high levels of social trust that come from living within a tight-knit, supportive social group. What the women were describing was consistent with the concept of fitness interdependence, an outcome of the (often passive) benefits of living in a social group. The formation of *pukurrpa* through costly sharing allows group members to live together and reduce the competition and stresses of social life that might tear groups apart. The formation of *pukurrpa* increases the likelihood not only that your hunting partner will share evenly with you, but also that when you divide labour it will be done cooperatively. For example, you can trust that when you leave your children behind, your friend will look after them, or that when you go hunting, your friend will get enough firewood to share with you when you return (and not just keep it for herself).

While these trust games require the other to pay a cost of varying degrees to repay that trust (expending energy getting wood, taking a cost to one's own foraging returns to care for children), others are much less costly. For example, one important trust game might occur if there are group augmentation benefits, such as deterrence from predators or raiding parties [7], and if group members trust their group-mates to stay and augment the group rather than leave to live elsewhere. People living in larger groups will be less vulnerable to threats from predators and outgroups, and ensuring that your group-mates are happy and not planning to leave would be one important outcome of generous sharing. In this case, the cost of betrayed trust (partner moves away) is quite high, and the partner's cost of repaying trust are very low (they do nothing different). In each context, the costs of betrayed trust will vary, e.g. a mother who comes back to lost or injured children experiences a much higher cost than one who returns to find that there is not enough wood collected to cook the meat she has hunted.

We propose that when one individual incurs a cost to help another, it signals how much the helper values the recipient (i.e. the helper's stake in the recipient), which increases levels of trust and allows each to benefit from that trust. For example, a good hunter could just attempt to profit from her own skill or work effort, but instead she shares with others to signal that she is dependent on them for the group augmentation benefits of being together. The signal keeps individuals together under the pressure of constant threats of competition and jealousy that cause groups to fission, which is particularly important in marginal environments where competition is intense but the benefits of cooperation are high. Thus, this signalling hypothesis explains the widespread unconditional sharing that anthropologists have often observed in hunter–gatherer societies [14–18], as well as other costs that humans incur to promote cooperation within courtships, friendships and religion [19–23]. We support our hypothesis with a formal model to show how costly acts can signal how much one values a recipient, when such signals will be honest and when they will increase trust. But first, we present the background theory on fitness interdependence and signalling to show how we arrive at this hypothesis.

### (a) Fitness interdependence, a.k.a. stake

Many organisms have a direct fitness stake in the welfare of other organisms. If A does something that benefits B—however incidental those benefits—then B has a vested interest in keeping A alive, well, and continuing to produce benefits for B. B's fitness is thus dependent upon A's fitness: when A does well, B does well (and sometimes *vice versa*, where interdependence is mutual dependence). As such, if B benefits from A's well-being, then B may unconditionally help A in order to ensure that A can continue to do whatever A does. This principle has been discovered multiple times and has been variously called stake [8], fitness interdependence [1], pseudo-reciprocity [24], byproduct reciprocity [25], partnership [26], group augmentation [7], irreplaceability [27] and vested interests [28]. Fitness interdependence does not require reciprocity: A just does what is in its own interest and need not even be aware about B. As long as A's actions happen to benefit B by any means—either within or outside of a reciprocal relationship—then B has some stake in A's welfare and has an interest in unconditionally helping, even if B's help is never observed [2]. Such fitness interdependence can be created in many ways, including genetic relatedness [5,6,8], affinal relations [29], long-term mating bonds, long-term reciprocal partnerships [2,27], group size effects [7] or byproduct benefits produced by one party [25]; the latter may include scrounging opportunities, learning opportunities or vigilance against a common enemy. The more interdependent people feel with each other, the more they cooperate [30].

Fitness interdependence is relationship-specific: you have a different stake in your spouse compared with that in your siblings, close friends, casual acquaintances and competitors, all of which are different from others' stake in those same people. Similarly, those different partners each have different stakes in you: your spouse and close allies value you highly, your casual acquaintances may be indifferent and your competitors may have a stake in your demise (negative fitness interdependence; [1]). Furthermore, interdependence can be asymmetric: you may depend upon someone more than they depend on you, such that you value them more than they value you. If you know someone has a stake in your welfare (or demise)—you know you can (or cannot) trust them to cooperate—then it changes your strategic response. Thus, it is important to assess who has a stake in your welfare, to know who will help you and repay your trust, and know who will harm you or betray your trust [27].

Furthermore, the interdependence between two individuals can change over time. Romantic and platonic relationships can become closer or more distant. One individual may soon leave the group, which gives them less stake in the welfare of other group members because they anticipate fewer future interactions [2]. Individuals may get better at producing benefits for others as they develop new skills (e.g. hunting ability), or may get worse as they senesce or shift their focus elsewhere (e.g. new parents have less time and surplus to share). New conflicts of interest can arise that give people an incentive to be spiteful instead of helpful [31]. Given that incentives change over time, one must constantly assess others’ stake in order to estimate who is most likely to cooperate (see also [32]).

If one person has a stake in another's welfare, this will affect how much the former values the latter in psychological terms [33,34]. For example, if A's fitness depends on B, then A will feel more warmth for B, more concern for B's welfare, more desire to help, and so on. A's valuation of B will thus track A's fitness stake in B, albeit imperfectly. This valuation can be quantified as their welfare trade-off ratio (WTR) towards each other [33,34]. WTR is a measure of how much you value another person, relative to how much you value yourself. For example, if A values B half as much as herself, then A's WTR towards B is 0.5, and A will help whenever the benefit (*b*) to B is more than twice the cost (*c*) to A. Formally, an organism will help whenever WTR × *b* > *c* [33], which is the same as Roberts's [8] formula for helping due to stake (*sb* > *c*, where *s* represents stake), which is itself a generalization of Hamilton's rule (*rb* > *c*, [5]) to non-genetic interests in partners. Studies show that WTR predicts people's willingness to cooperate (reviewed by [33,34]). People may attempt to change others' WTR towards themselves with such tactics as expressing gratitude for help received [35] or using anger to coerce the other person to alter their WTR [36]. People estimate others’ WTR based on the costs that those others incur to help [33,37].

### (b) Signalling of intent

How can organisms accurately assess how much others value their welfare, and what keeps such assessments accurate? Signalling theory is widely used to understand how signals can be honest despite some conflicts of interest between signallers and receivers (e.g. [38–41]). For signals to be honest, the benefits of signalling must outweigh the cost for honest signallers, but not for dishonest signallers. This can occur if honest signallers pay lower costs or receive higher benefits than dishonest signallers for a given signal. For example, good fighters are less likely to get injured (i.e. lower cost) in any given fight than poor fighters, so bellicosity can signal one's fighting ability—the signal is only worthwhile for good fighters who are less likely to lose [42,43]. Similarly, a hungry chick benefits more from food (i.e. higher benefits) than a sated chick, such that begging could be an honest signal of need that is only worthwhile for hungry chicks ([44]; though see [45]).

Most signalling models involve signals of quality or *ability*, e.g. a primate makes dominance displays to signal its fighting ability, or a wealthy person uses philanthropy to signal their wealth and ability to acquire resources [16,46]. However, signalling theory can also apply to signals of *intent*, i.e. a signal to prove that the signaller intends to follow through on a threat or promise [42,47,48]. Some models of intent just assume the differential costs for trustworthiness without specifying their ultimate source or why individuals differ (e.g. [49,50]). When specified, such signals of cooperative intent are maintained by the long-term benefits accruing to honest signallers: the signal costs are worthwhile for those who intend to reap the long-term benefits of mutual cooperation, but not for those who intend to engage in short-term defection [51] or leave before the interaction [52]. Thus, these signals of intent create trust between signaller and observer through signalling the actor's ‘shadow of the future’, that they will be around long enough to benefit from multiple rounds of cooperation. For example, costly apologies are more credible than ‘cheap talk’, because costly apologies are only worthwhile for someone who will not immediately revert to harming the other [20]. Similarly, costly courtship signals are worthwhile for suitors who intend to stay, but not worthwhile for suitors who intend to desert [19,23]. The costs of religious rituals are only worthwhile for those who intend to stay and cooperate [21,22]. Even cooperation in a generic Prisoner's Dilemma can be interpreted as a signal that one intends to be around long enough to benefit from reciprocation [32].

One of the most important signals of cooperative intent is generosity [21,53–56]. But not just any form of generosity builds the trust that enables cooperation: both empirical observations [12,14,57] and experimental work [58] suggest that costlier or more ‘genuine’ forms of generosity (e.g. distancing oneself from the status benefits) engender more trust than more selfish or aggrandizing forms of giving and helping. In the Martu case study described above, those who share a higher *proportion* of their income, not just larger *absolute* amounts, gain reputations for the generosity that lead to better access to partnerships for cooperative hunting. Such investments in another's well-being could function as an index of the value of the relationship (e.g. one's WTR for the recipient) and thus the level of interdependence, described by Martu as *pukurrpa*.

These theoretical frameworks and empirical case studies suggest a new mechanism for honest signalling of one's cooperative intent: signals that reveal an actor's stake in the recipient. By helping others, an actor signals that they value the recipient and thus have a vested interest in repaying the recipient's trust. Recipients gain useful information about the actor's stake and thus about the actor's incentives to cooperate. Signallers benefit from that trust, leading them to help at higher levels than they would otherwise be inclined (e.g. higher costs, worse benefit/cost ratios) in order to earn that trust. In other words, signals of stake may start out as a cue that observers attend to for their informational value, whereupon actors start actively signalling their stake in observers in order to be trusted (see [59], for a general argument on how cues can evolve into signals). In this paper, we provide a formal model of such a signalling system: actors with a stake in the recipient will signal that stake in order to be trusted, actors who do not value the recipient do not signal, and recipients attend to such signals to know whom to trust. We show the conditions under which such signalling systems are stable, and we show that the ‘helping’ acts need not benefit the recipient to function as signals of stake.

### (c) A note on terminology

Before presenting our model, we must clarify our terminology. The term ‘interdependence’ is used in two different senses: a broader definition including any way that two individuals affect each other (e.g. [30]), or the more specific ‘fitness interdependence’ whereby one organism's well-being affects another's well-being (e.g. [1]). We use the latter sense. *Inter*dependence is when two organisms are mutually dependent on each other (i.e. each has a stake in the other's welfare). Thus, we will use ‘stake’ for one organism's vested interest in another's welfare and will reserve ‘interdependence’ for when referring to the relationship as a whole. We will use the terms ‘stake’ and ‘valuation’ similarly, given that the latter is the psychological representation of the former and should track the former, albeit imperfectly (see [33,34]; electronic supplementary material). Thus, our model is a model of one organism signalling its valuation or stake in its partner, which is one side of an interdependent relationship. Readers who use these terms differently are invited to substitute their own terms for our concepts.

## 2. Model

### (a) Trust decision and signalling

**Figure 1.**
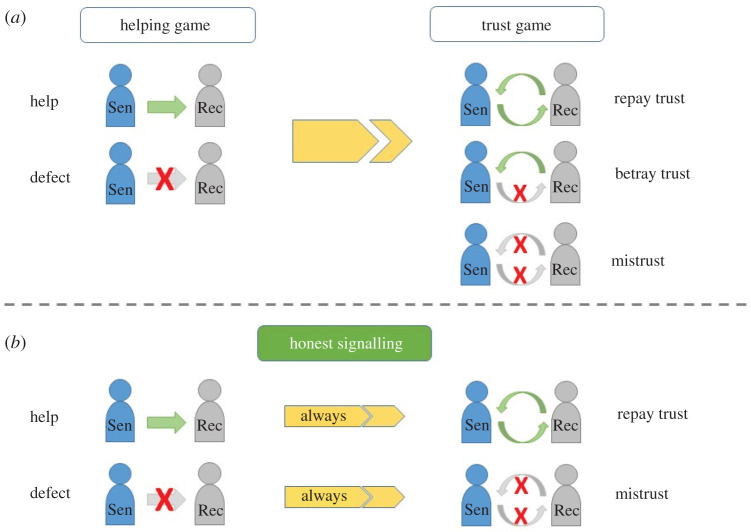
(*a*) Basic structure of the first game (helping game) and second game (trust game), and strategies for senders (Sen) and receivers (Rec). Whole green arrows without crosses indicate helping/trusting/repaying trust; arrows with a red X indicate a lack of helping/trusting/repaying trust. (*b*) Honest signalling equilibrium: senders who help always repay the trust and are therefore trusted; senders who defect will always betray the trust and are therefore not trusted. (Online version in colour.)

We model the above empirical problem in a two-stage process: a helping game (which serves as a signal) and a subsequent trust game. Imagine one agent (sender, abbr. Sen) has an opportunity to send a cooperative signal by helping another (receiver, abbr. Rec) at cost *c* to the sender and benefit *b* to receiver (the helping game). After getting the signal (i.e. receiving help), the receiver has the opportunity to trust the sender, whereupon the sender can either repay or betray that trust (trust game). Figure 1(*a*) describes the structure and strategies of these games. If the receiver does not trust, then both earn *P* (punishment for no trust). If the receiver trusts and the sender repays that trust, then both earn *R* (reward for trust repaid). If the sender betrays that trust, then the sender gets *T* (temptation to betray) and the receiver gets *S* (sucker's payoff). Assume that *R* > *P*.

### (b) Game types

If we standardize the payoffs for mutual cooperation and defection at *R* = 1 and *P* = 0, respectively [60], then trust situations differ only in the magnitude of the temptation to defect (*T*) and the sucker's payoff (*S*). Imagine a Martu hunter trusting others to perform a task while she hunts. Successful cooperation (*R*) results in more food or less effort than if she had to divide her time between both activities (*P*). There is a high temptation to defect (*T*) if the other person must perform an onerous task (e.g. collect heavy firewood) or has a high-payoff alternative activity to perform (e.g. sleeping), and the sucker's payoff (*S*) is poor if it is worse than mutual defection, especially if it imposes high costs on the hunter.

Rather than limit the model to situations with costly trust and reciprocation, we examine all four main possible game types. In the Prisoner's Dilemma, there is a big temptation to defect (*T* > *R*), and the worst outcome is to get suckered (*P* > *S*). For example, imagine a mother trusting her campmates to mind her child: child-minders have a big incentive to sleep (high *T*), and the betrayal of trust could have serious consequences (low *S*), like an unattended child falling into a campfire. Snowdrift Games also have a big temptation to defect (*T* > *R*), but the worst outcome is mutual defection (*S* > *P*), like the proverbial drivers stuck behind a snowdrift that neither will shovel. Such payoffs might be common in the division of labour contexts: imagine a hunter trusting others to gather wood for the cooking fire: the others have an incentive to slack or sleep (high *T*), and if they do not gather any wood, the hunter is better off gathering it herself rather than also defecting and having no fire (*S* > *P*). Stag Hunts have a high payoff for mutual cooperation (*R* > *T*) and a poor sucker's payoff (*P* > *S*). Such payoffs are common in cooperative hunting when a hunter focused on a single large prey item trusts their partner to stick to the ambush plan and not to get distracted by encounters with smaller animals. Finally, Harmony Games are associated with little incentive to defect because cooperating pays better regardless of what one's partner does (*R* > *T* and *S* > *P*). Imagine a village fire that could engulf all structures including one's own: cooperating to douse the flames is better than leaving it to a village-mate (*R* > *T*), but even acting alone is better than letting the fire blaze unchecked (*S* > *P*).

### (c) Value placed on partners (*V*)

Senders and receivers have some stake in each other's welfare (interdependence): the sender values the receiver's welfare by *V*_s_, and the receiver values the sender's welfare by *V*_r_ (the subscript states who is doing the valuing). *V*_s_ and *V*_r_ can range anywhere from −1 to 1: the sender's and receiver's fitnesses may be perfectly positively correlated such that they rise and fall together (*V* = 1), perfectly negatively correlated such that one's benefit is the other's detriment (*V* = −1), or completely uncorrelated such that they are perfectly ambivalent about each other's welfare (*V* = 0) (see [1] for positive and negative fitness interdependence; see [31,61] for positive and negative genetic relatedness; see [62] for altruistic versus spiteful preferences; see electronic supplementary material for additional ranges). A sender and a receiver thus represent two agents drawn from a population who could have any level of stake in each other. Each agent knows its own stake in the other, but not the other's stake in it—this is what it is trying to assess.

This stake gives senders an interest in helping receivers and in repaying their trust. Without stake, trusting depends only on whether *R* > *T*. Interdependence creates different stakes—different thresholds to repay the trust (see electronic supplementary material, figures S1 and S2), thus it creates variability in senders' behaviour. In turn, this variability gives an incentive for the receiver to find out how much the sender values them (i.e. *V*_s_). We test whether the sender's behaviour in the first (helping) game could honestly signal their expected behaviour in the second (trust) game. For example, to convince a mother of her trustworthiness while that mother gathers wood, a potential child-minder could give some food to the mother, at cost *c* to the child-minder and benefit *b* to the mother. The more food given, the higher the cost *c* and benefit *b*. The more valuable the food or the favour to the mother, the higher the benefit *b*.

In the first game (helping game), this stake functions exactly as in Roberts [8]: a sender will unconditionally incur cost *c* to confer benefit *b* upon a receiver when *bV*_s_ > *c*, which is the same as Hamilton's rule in biology ([5]; *br* > *c*, where *r* denotes relatedness). However, the sender may sometimes also send the signal when *bV*_s_ − *c* < 0 because the signal causes the receiver to trust—it is these signals that we investigate here. Electronic supplementary material, figure S1 shows that, for all values of *c* and *b*, senders help more when signalling is possible than when helping is unconditionally based on stake alone. Notably, when signalling is possible, the sender often helps even if it values the receiver's demise (*V*_s_ < 0), if the costs of helping outweigh the benefits to the receiver (*c* > *b*), or even if there are no benefits (*b* = 0)

Would such signals be honest? An honest equilibrium is defined by a correlation between the behaviour in the helping game and the behaviour in the trust game. At a fully honest equilibrium, those individuals helping in the first game (i.e. giving a signal) will always repay trust in the second game; those who would betray will not help (figure 1*b*). What are the conditions for such honest equilibria? Note that a sender's quality is defined by their trust game behaviour: trustworthy senders will repay trust, untrustworthy senders will betray. Since trustworthiness is binary in this simple game, a binary signal is enough; i.e. receivers are not interested in the amount given in the first game, only whether help was provided or not.

### (d) Decisions and payoffs

**Figure 2.**
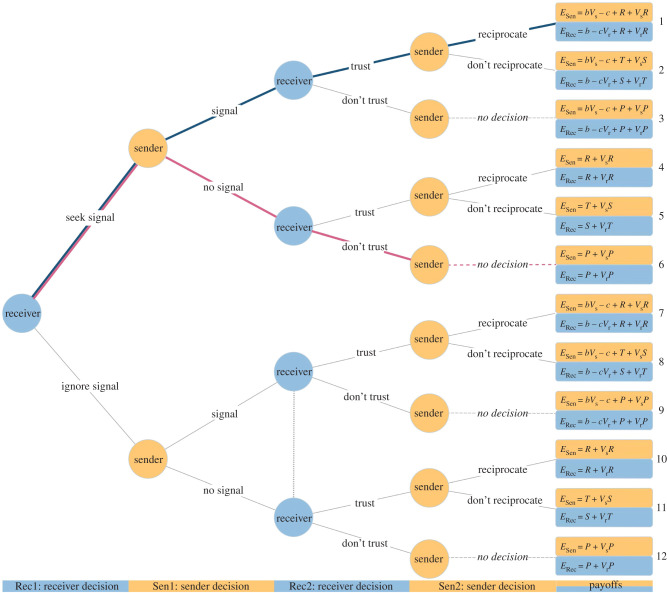
The structure of the game for senders (Sen) and receivers (Rec). Orange and blue nodes indicate decisions made by the sender and by the receiver, respectively. Numbered terminal nodes on the right denote the potential outcome of the game; each terminal node assigns a payoff to the sender (*E*_Sen_) and to the receiver (*E*_Rec_) depending on the decisions leading to the terminal node. The blue and red paths (nodes 1 and 6) represent the pathways for trustworthy and untrustworthy senders, respectively. The dotted vertical line in Rec2 represents informational symmetry, i.e. the receiver is unaware of the state of the signaller.

All in all, there are **four** decisions: (i) listen to signals or ignore them (receiver's Rec1 decision); (ii) signal versus not signal, i.e. help in the helping game by paying cost *c* to confer benefit *b* (sender's Sen1 decision); (iii) trust versus do not trust in the trust game (receiver's Rec2 decision); and (iv) repay trust versus not repay trust (sender's Sen2 decision). If receivers do not trust, then senders have no opportunity to repay or betray, leaving 12 possible outcomes (nodes). The fitness of the sender (*E*_Sen_) and the recipient (*E*_Rec_) is the sum of the payoffs from the helping and trust game respectively. Figure 2 shows the corresponding extensive form game and payoffs.

### (e) Analysing the honest equilibrium

At the honest equilibrium: (i) senders with a high stake will signal (help in the first game), receivers will select senders (i.e. trust them) based on their signal, and senders will repay the trust; (ii) senders with a low stake will not signal, will not receive the trust and thus have no further decision to make. These two outcomes represent nodes 1 and 6, respectively, in figure 2, so to find the honest equilibrium, we need to solve the pathway that leads to these nodes. To do so, we will use ‘backward induction’ [63] and sequentially solve for (i) the sender's optimal Sen2 decision of whether to repay the receiver's trust; (ii) the receiver's optimal Rec2 decision of whether to trust the receiver, knowing the sender's optimal Sen2 decision; (iii) the sender's optimal Sen1 decision as to whether to send a signal (i.e. pay *c* to confer benefit *b*), knowing the receiver's optimal Rec2 trust decision; (iv) the receiver's optimal Rec1 decision as to whether to demand signals, knowing the sender's optimal Sen1 signalling decision. Details of this process can be found in the electronic supplementary material; here we report the conditions that need to hold in order for the pathway leading to node 1 to be evolutionarily stable (for more on evolutionarily stable strategies, see [64]).

#### (i) Step 1. Sender's Sen2 decision to repay or not repay trust (terminal nodes 1 versus 2)

Senders will repay trust when their payoff at node 1 is higher than at node 2 (figure 2), which simplifies to:

(Inequality 1) $$V_{s}>\frac{T-R}{R-S}.$$

As such, the sender's stake increases the probability that the sender will repay the trust; hence it is in the receiver's interest to gain information about the sender's stake. Senders will repay trust more often when there are high rewards for mutual cooperation (*R*), when the temptation to defect (*T*) is low, and if *V*_s_ > 0, and when the sucker's payoff (*S*) is low. Note: Inequality 1 holds for not just node 1 versus node 2, but any repayment decision (i.e. node 4 versus 5; node 7 versus 8; node 10 versus 11)

#### (ii) Step 2a. Receiver's Rec2 decision to trust the sender with a signal (terminal nodes 1 versus 3)

Receivers will trust a trustworthy signaller when their payoff is higher at node 1 than node 3 (figure 2), which simplifies to:

(Inequality 2) $$V_{r}>\frac{P-R}{R-P}.$$

Because (*P* − *R*)/(*R* − *P*) = −1, this condition will always hold as long as *V*_r_ > −1. In other words, the receiver will always trust the sender who gave a signal. Note: Inequality 2 holds for any comparison of trusting versus not-trusting a trustworthy sender (i.e. node 1 versus 3; node 4 versus 6; node 7 versus 9; node 10 versus 12).

#### (iii) Step 2b. Receiver's Rec2 decision to trust the sender without a signal (terminal nodes 5 versus 6)

Receivers will distrust an untrustworthy signaller when their payoff is higher at node 6 than node 5 (figure 2), which simplifies to

(Inequality 3) $$V_{r}<\frac{P-S}{T-P}.$$

This further simplifies to *V*_r_ < − *S*/*T* (when *P* = 0). This condition is likely to hold when there is a poor sucker's payoff (i.e. *S* < 0) and the temptation to betray is low. Note: Inequality 3 holds for any comparison of trusting versus not-trusting an untrustworthy sender (i.e. node 2 versus 3; node 5 versus 6; node 8 versus 9; node 11 versus 12).

#### (iv) Step 3a. Trustworthy sender's Sen1 decision to signal (terminal nodes 1 versus 6)

Trustworthy senders will signal (i.e. help the receiver) when their payoff is higher at node 1 than node 6 (figure 2), which simplifies to

(Inequality 4) $$V_{s}(b+R-P)+(R-P)>c.$$

In other words, senders will send a signal when they highly value the receiver (*V*_s_), when gains from the signal (*b*) are high, and when the gains from cooperation (*R* − *P*) are high.

#### (v) Step 3b. Untrustworthy sender's Sen1 decision not to signal (terminal nodes 2 versus 6)

Untrustworthy senders will refrain from signalling (i.e. helping the receiver) when their payoff is higher at node 6 than node 2 (figure 2), which simplifies to

(Inequality 5) $$V_{s}(b+S-P)+(T-P)<c.$$

In other words, untrustworthy senders will refrain from signalling if they do not value the receiver (*V*_s_), when the no-trust payoff (*P*) is decent, and when the temptation to defect (*T*) and sucker's payoff (*S*) are low.

**Figure 3.**
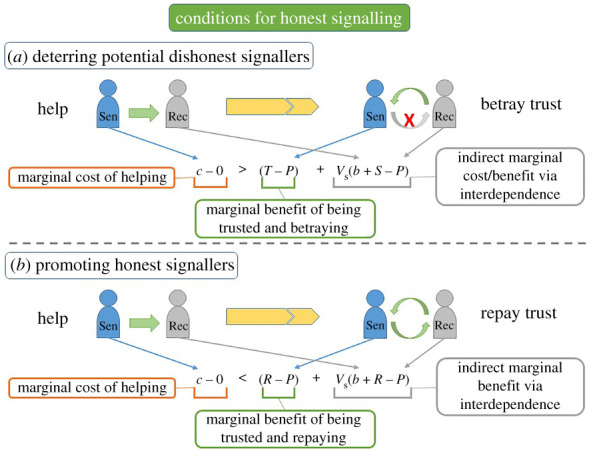
Conditions for honest signalling and where the costs and benefits come from. Helping costs *c* to the sender (Sen) and provides benefits *b* to the receiver (Rec); the sender values the receiver's payoff by *V*_s_. If there is no trust, then senders and receivers both earn *P*. (*a*) If a dishonest sender pays to help but then betrays the trust, they receive *T* (instead of *P*), and the receiver earns *b* + *S* (instead of *P*). Thus, a dishonest sender will not help when the cost of helping outweighs their marginal benefit of being trusted and betraying that trust plus the harm inflicted on the receiver, i.e. when *c* − 0 > (*T* − *P*) + *V*_s_ (*b* + *S* − *P*). (*b*) If an honest sender helps and then repays the trust, they receive *R* (instead of *P*), and the receiver earns *b* + *R* (instead of *P*). An honest sender will help when the cost of helping is less than their marginal benefit of being trusted and repaying that trust plus their stake in the receiver, i.e. when *c* − 0 < (*R* − *P*) + *V*_s_(*b* + *R* − *P*). (Online version in colour.)

If we let *V*_st_ and *V*_su_ be the values placed on the beneficiary by trustworthy and untrustworthy senders, respectively, then we can combine Inequalities 4 and 5 to show that signals will be honest when (figure 3):

(Inequality 6) $$\begin{array}{rl} V_{\mathrm{st}} & \left(b+R-P)+(R-P)>c>V_{\mathrm{su}}(b+S-P\right)\\ & +(T-P). \end{array}$$

#### (vi) Step 3c. Calculating the signal cost threshold

From the above conditions, we can calculate the signal cost (donation in the first game) that is guaranteed to be honest, i.e. a signal cost that only trustworthy senders will pay. From Inequality 6 (also figure 3), untrustworthy senders will not signal when *c* > *V*_su_(*b* + *S* − *P*) + (*T* − *P*). For a completely ambivalent sender (*V*_s_ = 0), this simplifies to *c* > *T* − *P*.

However, there are two other types of untrustworthy senders: (i) ‘weakly interdependent senders' who value the receiver, but not enough to repay their trust (i.e. 0 < *V*_s_ < (*T* − *R*)/(*R* − *S*)); and (ii) ‘hostile senders’ who place negative value on the receiver's welfare (i.e. *V*_s_ < 0), e.g. senders who compete with the receivers for valuable resources. To be honest, signals must be costly enough to deter each of these types. These types are often harder to deter than ambivalent senders (i.e. *V*_s_ = 0): hostile senders are incentivized to signal by receivers' losses (i.e. when *b* + *S* − *P* < 0) and are deterred by receivers’ gains; weakly interdependent senders are incentivized to signal by receivers' gains (i.e. when *b* + *S* − *P* > 0) and are deterred by receivers’ losses.

The level of cost that will deter all untrustworthy senders is

(Inequality 7) $$\begin{array}{r} c>T-S-b\; \end{array}$$

(when *b* + *S*−*P* < 0, i.e. net loss) and

(Inequality 8) $$\begin{array}{rl} & c>(T+P)+(T-R)\frac{b+S-P}{R-S}\\ & (\mathrm{when}\;b+S-P>0,i.e.\;\mathrm{net}\;\mathrm{gain}). \end{array}$$

**Figure 4.**
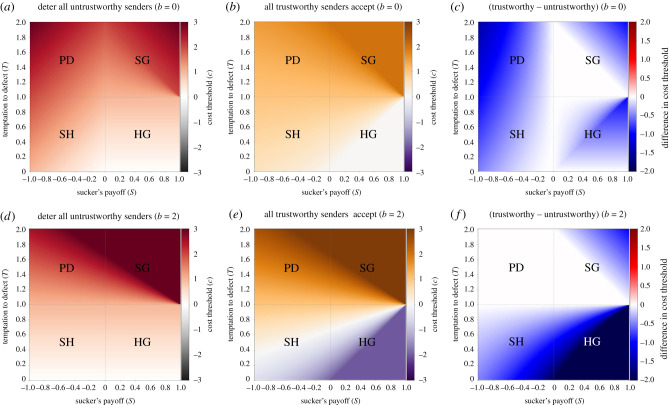
Costs of honest signals of fitness stake: top row *b* = 0 (*a*–*c*), bottom row *b* = 2 (*d*–*f*); (*a*,*d*) cost of honest signals that deter all untrustworthy senders (left-hand side of Inequality 6); (*b*,*e*) cost of signals that all trustworthy senders are willing to pay (right-hand side of Inequality 6); (*c*,*f*): difference between the two, where blue regions show where the first is larger than the second. Parameters are *R* = 1, *P* = 0. From left to right and top to bottom, the four quadrants represent parameter values for Prisoner's Dilemmas (PD: *T* > 1, *S* < 0), Snowdrift Games (SG: *T* > 1, 0 < *S* < 1), Stag Hunts (SH: 0 < *T* < 1, *S* < 0) and Harmony Games (HG: 0 < *T* < 1, 0 < *S* < 1).

If receivers experience a net zero from the exploitative interaction (i.e. *b* + *S* − *P* = 0), then these all simplify to *c* > *T* − *P*. Figure 4 shows the minimal cost for each combination of *S* and *T* that would deter all types of dishonest senders from signalling. The electronic supplementary material presents the detailed derivation.

Figure 4*a*,*d* shows that low-cost signals can often be honest, especially when the temptation to defect *T* is less than the reward for mutual cooperation *R* (e.g. Harmony Games, Stag Hunts). In such cases, there is little incentive for the sender to defect unless she is hostile to the receiver (*V*_s_ < 0), especially at higher *b*. As the temptation to defect (*T*) increases, the costs must be higher to deter untrustworthy senders. However, not all trustworthy senders are willing to pay those signal costs. Figure 4*b*,*e* shows the maximum costs paid by the least-willing trustworthy sender; Figure 4*c*,*f* shows that these costs are often less than what the most-willing untrustworthy sender would pay. The white areas of Figure 4*c*,*f* thus represent a fully separating equilibrium where all trustworthy senders signal and no untrustworthy senders do; the blue areas represent a partial separating equilibrium where either some trustworthy senders are deterred from signalling *or* some untrustworthy senders will signal. Thus, the depth of blue in figure 4*c*,*f* is an index of how much dishonesty or deterrence there is in the signalling system.

Which trustworthy senders will pay the signal costs that deter *all* untrustworthy senders, i.e. the signal costs that are guaranteed to be honest (henceforth ‘guaranteed signals’)? Inequality 4 (figure 3) says that honest signallers will pay the cost when *V*_s_(*b* + *R* − *P*) + (*R* − *P*) > *c*. We can substitute this into the costs in Inequalities 7 and 8 to get the following:

Condition 1: when *b* + *S*−*P* < 0 (i.e. net loss to receivers), trustworthy senders will pay *T*−*S*−*b* when

(Inequality 9) $$V_{s}>\frac{T-S}{b+R-P}-1.$$

Condition 2: when *b* + *S* − *P* > 0 (i.e. net gain to receivers), trustworthy senders will pay (*T* − *R*)(*b* + *S* − *P*)/(*R* − *S*) + (*T* − *P*) when

(Inequality 10) $$V_{s}>\frac{T-R}{R-S}.$$

**Figure 5.**
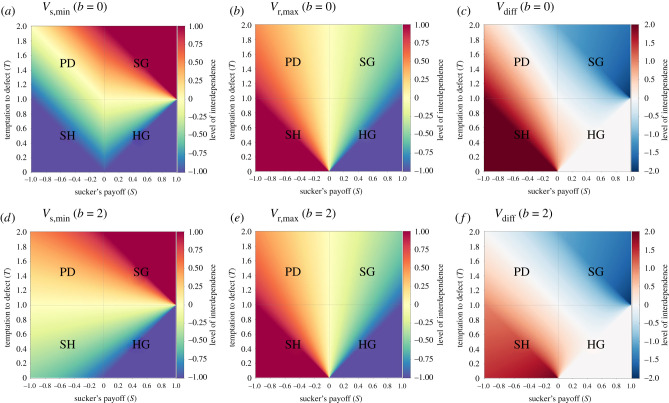
Conditions for honest signals; (*a*,*d*) sender's minimum stake in the receiver (*V*_s,min_) to be willing to pay the signal costs that deter all untrustworthy senders; solid blue (red) areas represent parameter regions where senders always (never) pay the signal costs; (*b*,*e*) receiver's maximum stake in the sender (*V*_r,max_) to demand signals instead of trusting blindly; solid red (blue) regions are where receivers will always (never) demand signals; (*c*,*f*) regions where signalling systems are more likely (red) or less likely (blue) to exist; values displayed are *V*_diff_ = *V*_r,max_ − *V*_s,min_. Signalling will occur when *V*_r,max_ > *V*_s,min_ (red areas), could occur when these stakes are asymmetric but similar in value (light blue areas) and is unlikely to occur when *V*_s,min_ ≫ *V*_r,max_ (dark blue areas); (*a*–*c*): *b* = 0; (*d*–*f*): *b* = 2. All panels: parameters are *R* = 1, *P* = 0. From left to right and top to bottom, the four quadrants represent parameter values for Prisoner's Dilemmas (PD: *T* > 1, *S* < 0), Snowdrift Games (SG: *T* > 1, 0 < *S* < 1), Stag Hunts (SH: 0 < *T* < 1, *S* < 0) and Harmony Games (HG: 0 < *T* < 1, 0 < *S* < 1).

Figure 5*a*,*d* graphs these as the minimum stake *V*_s_ that senders must have in the receiver in order to be willing to pay those guaranteed signalling costs; we call this *V*_s,min_.

#### (vii) Step 4. Receiver's Rec1 decision to search for signals

Here the receiver must decide whether to look for signals before making a decision in the trust game. When the receiver ignores signals, she has no information on which to base her trust decisions, so she cannot distinguish between nodes with signals (nodes 7–9, figure 2) or without (nodes 10–12). An ignorant receiver thus has three options: (i) reject everyone, (ii) trust everyone and have her earnings depend on whether the sender is trustworthy, or iii) randomly pick either rejecting or trusting, which is the weighted sum of (i) and (ii) and thus never better than both, so we will ignore it. Let us assume that the frequencies of sender with high stake (*V*_H_) who will repay the trust, and low stake (*V*_L_) who will betray the trust, are in proportions *q* and 1 − *q*, respectively. Let *E*_r,signals_, *E*_r,reject_ and *E*_r,accept_ denote the payoffs for receivers attending to signals, rejecting everyone and accepting everyone, respectively. The following inequalities describe the payoffs for these choices:

(Equation 1) $$E_{r,\mathrm{signals}}=q(b-cV_{r}+R+V_{r}R)+(1-q)(P+V_{r}P),$$

(Equation 2) $$E_{r,\mathrm{reject}}=q(b-cV_{r}+P+V_{r}P)+(1-q)(P+V_{r}P)$$

(Equation 3) $$E_{r,\mathrm{accept}}=q(b-cV_{r}+R+V_{r}R)+(1-q)(S+V_{r}T).$$

Combining these, looking for signals pays better than blindly not-trusting everyone when

(Inequality 11) $$V_{r}>\frac{P-R}{R-P},$$

which is true for all *V*_r_ > − 1. Looking for signals pays better than blindly trusting when

(Inequality 12) $$V_{r}<\frac{P-S}{T-P}.$$

Combining the two above conditions gives

(Inequality 13) $$\frac{P-S}{T-P}>V_{r}>\frac{P-R}{R-P}.$$

In other words, receivers will demand signals unless they care enough about the sender that they do not mind getting suckered by them, which occurs when *S* is a decent payoff and/or *T* is big enough for a receiver they value.

Figure 5*b*,*e* shows the threshold where receivers switch from demanding signals (low *V*_r_) to trusting blindly (high *V*_r_); this is the highest level of *V*_r_ when signals will be demanded (call it *V*_r__max_). Figure 5*b*,*e* shows that it is often beneficial for the receiver to be choosy in the Prisoner's Dilemma and the Stag Hunt. By contrast, in the Snowdrift or the Harmony Game, the sucker's payoff is better than mutual defection (i.e. *S* > *P*), so there is no risk to blindly trusting. As such, in the Snowdrift or Harmony Game, receivers will only demand signals if they want to avoid having the sender benefit (i.e. if *V*_r_ < 0).

### (f) Summary of steps 1–4

Table 1 summarizes the conditions under which each requirement is met.

**Table 1.** Main conditions of honest signalling for senders and receivers.

stage	question	condition	comment
Sen2 trust game	sender repays trust if	*V*_s_ > (*T*−*R*)/(*R*−*S*)	always true in Harmony Game and Stag Hunt (*T* < *R*) unless *V*_s_ is negative. If *S* > *T* and *T* < *R*, then sender will always repay trust regardless of *V*_s_
Rec2 trust game	receiver trusts signaller if	*V*_r_ > (*P*−*R*)/(*R*−*P*)	true whenever *V*_r_ > −1
Sen1 helping game	(i) trustworthy sender gives if	(i) *V*_s_ > (*c* − *R* + *P*)/ (*b* + *R*−*P*)	by comparison, giving only occurs without signals if *V*_s_ > *c*/*b*
	(ii) deceptive sender does not give if	(ii) (*c* – *T* + *P*)/ (*b* + *S*−*P*) > *V*_s_	
Rec1 helping game	(i) looking for signals instead of blind trust	(i) *V*_r_ < (*P*−*S*)/(*T*−*P*)	(i) only true in Snowdrift and Harmony Games (*S* > *P*) if *V*_r_ is negative
	(ii) looking for signals instead of blindly rejecting everyone	(ii) *V*_r_ > (*P*−*R*)/(*R*−*P*)	(ii) true whenever *V*_r_ > −1

### (g) When will signalling systems exist?

A signalling system will exist when receivers demand signals (figure 5*b*,*e*) and only honest signallers are willing to pay the cost to signal (figure 5*a*,*d*). A signalling system can thus exist whenever there are some receivers who value senders little enough to still demand signals (*V*_r_ < *V*_r,max_), yet some senders who value receivers highly enough to pay the signal costs (*V*_s_ > *V*_s,min_). If we assume that interdependence is symmetric, then this will occur under any conditions where *V*_r,max_ > *V*_s,min_. In such cases, signalling will occur within pairs with medium interdependence (i.e. *V*_r,max_ > *V* > *V*_s,min_). Highly interdependent pairs will have no signalling because receivers do not demand signals (*V*_r_ > *V*_r,max_), whereas weakly interdependent pairs will have no signalling because senders are unwilling to pay the costs (*V*_s_ < *V*_s,min_).

When interdependence is asymmetric, signalling systems can also occur under broader conditions (e.g. *V*_r,max_ < *V*_s,min_) if the sender values the receiver more than *vice versa*. In such cases, a highly dependent sender may be willing to send signals to a weakly dependent receiver who demands signals (see electronic supplementary material for a discussion of asymmetric interdependence). However, this is only biologically plausible when *V*_r,max_ and *V*_s,min_ are similar in value: if *V*_s,min_ >> *V*_r,max_, not many real-life pairs will have interdependences asymmetric enough such that a sender will be so dependent (*V*_s_ > *V*_s,min_) on a receiver who values them so little as to demand signals (*V*_r,max_ > *V*_r_).

Figure 5*c*,*f* shows the conditions where signalling systems are likely, either because *V*_r,max_ > *V*_s,min_ (red regions) or because *V*_r,max_ and *V*_s,min_ are close enough in value that asymmetric interdependence can result in some signalling; the darker blue the parameter space, the less likely that there will be signalling. Figure 5*c*,*f* reveals that signalling systems are likely in Stag Hunts, Harmony Games and Prisoner's Dilemmas, but not in Snowdrift Games.

## 3. Discussion

We find that acts of helping can function as signals of stake or valuation of a partner. Senders who value the receiver will pay to signal their incentive to repay the receiver's trust, whereas the cost of such signals will deter senders who will not repay the trust. The trust towards honest signallers then leads to better outcomes for both parties. Interestingly, signalling can result in senders helping under conditions where they would not normally help (i.e. without signalling), such as when the help costs the sender more than it benefits the receiver (*c* > *b*) or from slightly hostile senders in Stag Hunts (figure 4*b*,*e*; electronic supplementary material, figure S1). Our model shows that the ‘help’ need not even provide any real benefit to the receiver—it can still signal the sender's willingness to cooperate at *b* = 0. This matches existing models of courtship signalling, where a suitor may give costly but useless gifts to signal his intention to stay instead of desert (as emphasized by the article title ‘Why buy your darling flowers' [19]; see also [23]).

We see different conditions for signalling emerge when we introduce the possibility of conflict and competition in social interactions by allowing stake to be negative, such that some individuals benefit from harming others. Without such negative stakes, signalling is only favoured when the trust scenario is a Prisoner's Dilemma (i.e. a high temptation to defect and poor sucker's payoff, like sleeping versus watching a toddler). Without negative stake, no signalling is needed in Harmony Games or Stag Hunts (e.g. extinguishing village fires and cooperative hunts, respectively) because the sender has little incentive to defect, and no signalling is needed in Harmony or Snowdrift Games (e.g. gathering firewood) because the receiver is willing to get suckered rather than face mutual defection. However, this situation changes once some group members do better when others do worse: senders might spitefully defect because it hurts the receiver more than themselves, and receivers must be wary of that. When such a negative stake is possible, we find cooperative signalling in each of the four types of trust games: Prisoner's Dilemmas (e.g. watching toddler), Snowdrift Games (e.g. gathering wood), Stag Hunts (e.g. cooperative hunting) and Harmony Games (e.g. extinguishing village fires). According to our model, even those who are slightly hostile to each other may nevertheless repay the trust (in Stag Hunts and Harmony Games), and slightly hostile senders may signal to prove that they do not hate the receiver *quite* enough to spitefully defect.

Thus, our model predicts that signalling is more likely under conditions where an individual has at least some possibility of negative interdependence with at least one other individual. As such, we might expect to find costlier forms of generosity signalling where communities are more fluid and residentially mobile, conflict and competition are more common, and individuals reside with those with whom they might be competitors. Signalling systems should be more widespread if one must sometimes cooperate with one's competitors, compared with when individuals positively assort (or reside) only with those who share positive fitness interdependence.

In our model, different situations result in different signal costs. Signals can be extremely cheap when senders have little incentive to defect (i.e. Harmony Games and Stag Hunts)—this is like ‘cheap talk’ that allows parties to coordinate on mutually beneficial outcomes [65,66]. By contrast, signals may need to be expensive when the sucker's payoff is decent (i.e. Snowdrift Games). When the sucker's payoff is decent, senders will be less deterred by suckering someone they care about, so signal costs must be higher to deter dishonest signals; in practice these signal costs are often too high to be worthwhile for senders. Based on the different signalling costs in different situations, we can predict that signallers who consistently face certain kinds of cooperative dilemmas might invest more in sending costlier signals. For example, in the Martu case, women often face Snowdrift or Prisoner's Dilemma type trust contexts, such as child-minding, or divisions of labour where the tasks that must be performed are onerous and energetically expensive. Martu men, however, rarely face such cooperative dilemmas; they are most likely to cooperate (in subsistence) with each other primarily in the context of hunting kangaroo, which may be more of a Stag Hunt or even Harmony Game. Our model may thus explain why Martu women are more likely to pay a higher cost when sharing small animals than men [12], why hunters in many societies often distance themselves from personally distributing their catch, and more generally, why some types of individuals pay higher costs to help others.

Another important strength of our model is that it only requires a single two-stage interaction—signal and trust game—without need for further interaction or direct reciprocity between signaller and observer after the trust game. Other models require future interactions to make the signals worthwhile for cooperators (e.g. [19,20,23,32,51,52]). By contrast, if you value someone's welfare, future rounds are not necessary. Interdependence can arise from future reciprocity [2], but also in other ways. For example, kin have a stake in each others' welfare even if they will never interact again (e.g. pre-dispersal). Without kinship, our model requires that the sender depends on the receiver in some way in the future, but direct interaction is not necessary. For example, group-living organisms may value group members who will watch for predators or get eaten in their stead [7], villagers have a stake in the welfare of soldiers who will go to fight invaders even if the soldiers never return to that village, and people may value their distant in-laws who look after mutual kin (e.g. a sister-in-law whose children are one's nieces and nephews). Our model can account for all signals of stake regardless of the time horizons involved.

Furthermore, it is easy to understand how such signals of stake can evolve. Biernaskie *et al*. [59] show that signals can start out as cues: if a cue is correlated with some hidden trait, observers will start attending to that cue, whereupon it pays to invest in actively signalling that trait. Signals of stake can arise this same way. When senders have a stake in receivers' welfare, they have an incentive to unconditionally help. Receivers can then start attending to that helping and basing their trust on it, whereupon senders will invest in actively signalling their stake by helping under much wider circumstances (including *b* = 0). Barclay & Barker [67] made a similar argument with pro-environmentalism: they provide a simple mathematical model of how pro-environmental behaviour can be a cue of how much the actor values people who benefit from that environment; they also provide empirical evidence that pro-environmentalism does function as an honest signal of cooperative intent and is treated as such. Our model is more general in that it shows how senders will actively signal their stake, when such signals will be given and be honest, and when receivers will demand signals.

One limitation of our model is the use of binary helping, trust and reciprocation. In real life, these may be continuous variables, e.g. not *whether* you help someone, but how *much*. Our assumption of binary signals is not problematic if the beneficiary's trust decision is binary, because binary signals are sufficient for binary decisions—receivers can dichotomize a non-binary signal by setting a threshold, and only believing signals that exceed that threshold. Non-binary signals are only required for non-binary trust decisions (i.e. how *much* to trust someone); future work should examine non-binary helping, trust and reciprocation decisions.

Overall, our model provides a general framework for when helpful behaviour will be used to signal one's valuation of others and thus one's willingness to repay their trust. These signals can be worthwhile even between hostile parties or when the signal provides no benefit to the recipient. Signals are more likely to be used when one must distinguish oneself from hostile parties. Future work should investigate how such stake develops, how cues of stake evolve into signals and how the signalling is affected by different time courses, socio-ecological contexts and cooperative dilemmas. We look forward to empirical tests of our predictions.

## Data accessibility

Model details can be found in the electronic supplementary material.
